# Fatigue Indices and Perceived Exertion Highlight Ergometer Specificity for Repeated Sprint Ability Testing

**DOI:** 10.3389/fspor.2020.00045

**Published:** 2020-05-15

**Authors:** Hugo A. Kerhervé, David G. Stewart, Chris McLellan, Dale Lovell

**Affiliations:** ^1^Univ Rennes, M2S - EA 7470, Rennes, France; ^2^School of Health and Sport Sciences, University of the Sunshine Coast, Maroochydore, QLD, Australia; ^3^Faculty of Health Sciences and Medicine, Bond University, Gold Coast, QLD, Australia; ^4^School of Health and Wellbeing, University of Southern Queensland, Toowoomba, QLD, Australia

**Keywords:** anaerobic capacity, aerobic power, non-motorized treadmill, exercise mode, exhaustion

## Abstract

This study aimed to compare the time course of measures of performance, fatigue, and perceived exertion during repeated-sprint ability (RSA) testing performed on a non-motorized treadmill (NMT) and cycling ergometer (CE). Fourteen physically active participants performed two 10 ×6 s^−1^ RSA tests with a 1:4 work-to-rest ratio (24 s recovery) on NMT and CE. Measures of performance [peak and mean power output (PPO and MPO), cadence, and the time to reach PPO (TTP)] and of fatigue (fatigue index and decrement score) and ratings of perceived exertion (RPE) were collected during each session. The level of significance was set at *p* < 0.05. Participants completed the RSA test at a MPO of 1,041 ± 141 W on CE and 431 ± 48 W on NMT, achieving PPO of 2,310 ± 339 W on CE and 1,763 ± 289 W on NMT. Participants' weight was significantly correlated with PPO and MPO on CE (*p* < 0.001) and with MPO on NMT (*p* < 0.001). PPO on CE and NMT was significantly correlated only for absolute measures of power (*p* < 0.01). Cadence was higher and decreased throughout the RSA on NMT compared to CE, where it decreased only at the seventh bout. TTP was significantly shorter and more affected by fatigue on NMT than on CE. Fatigue indices were significantly greater on NMT compared to CE, with significant correlations between the decrement score and absolute and relative PPO on CE and NMT, between the fatigue index and absolute and relative PPO only on NMT, and no significant correlations with MPO. During RSA, RPE increased more on NMT compared to CE from bouts 3 to 7. During recovery, RPE was consistently higher on NMT at 1, 3, and 5 min post exercise compared to CE. These findings indicate that RSA performed on NMT induces greater fatigue and physiological load than CE, which originated in the lower resistive torque typically used on NMT compared to CE, resulting in a front loaded power output profile from the greater acceleration and cadence. From these results, we discuss that despite providing highly correlated measures of power output, NMT and CE should not be used interchangeably to assess RSA as they elicit markedly different responses. We also discuss these results from the fundamental differences in active muscle mass and power application patterns between running and cycling, which could form the basis of future studies.

## Introduction

Conditioning athletes for specific physiological, metabolic, mechanical, and psychological demands met in competition is a fundamental principle of athletic training. A popular form of training for the intermittent nature of field-based sports relies on enhancing repeated-sprint ability (RSA), using exercises characterized by short intervals (up to 10 s) performed at or near maximal abilities and repeated with incomplete rest (up to 60 s; (Girard et al., 2011)). The evaluation of RSA for field-based sports is commonly carried out either in field or laboratory settings. Overall, field running repeated sprinting is preferable, as it has been shown to be more reliable (Hopkins et al., 2001) and more specific (Bishop et al., 2001) for RSA testing of athletes in field-based sports compared to similar exercises using ergometry because of the task dependency of fatigue (Girard et al., 2011) and physiological systems (Bishop et al., 2011). Still, RSA testing in indoors settings using ergometers is commonly used when field measures are not available, or for the measure of variables not easily implemented in the field (power, force–velocity profiling).

Cycling ergometers (CE) have been routinely used for RSA testing as they allow rapid acceleration and self-determination of pace during short bouts of exercise performed at or near maximal abilities. Developed over three decades ago (Lakomy, 1987), non-motorized treadmills (NMT) have become increasingly popular for the same reasons. The reliability of measures used in power sports applications provided by flat-surfaced and curved designs of NMTs has been well-established against overground sprinting (Highton et al., 2012), CE (Chia and Lim, 2008; Gonzalez et al., 2013), or on separate days (Tong et al., 2001; Hughes et al., 2006) even with minimal familiarization (Glaister et al., 2007). Consequently, NMTs are now used along with CE for RSA testing (Sutton et al., 2000; McLain et al., 2015; Tofari et al., 2015) and used to simulate specific demands of field-based sports (Carling et al., 2012; Nédélec et al., 2013; Aldous et al., 2014).

However, while we know that task specificity likely influences the nature and magnitude of fatigue development (Girard et al., 2016), the effect of ergometer on RSA exercise has not been explicitly studied and there is still a paucity of applied sports literature to guide practitioners through ergometer selection and justification. The evaluation of NMT reliability has so far emphasized discrete measures of peak and mean power output performed during 1 × , 2 × , or 3 × repetitions of ~6s to 30 s sprints sprints with long (~2 min) recovery (Tong et al., 2001; Lim and Chia, 2007; Chia and Lim, 2008; Highton et al., 2012; Gonzalez et al., 2013), and only one study has used a RSA exercise (6 ×6 s^−1^), which induced only mild (~10%) performance decrements (Hughes et al., 2006). Longer RSA tests inducing marked performance decrements have commonly been used in research to study energy pathways contributions (Gaitanos et al., 1993), neuromuscular function (Billaut et al., 2005, 2006; Mendez-Villanueva et al., 2008), as well as the effect of recovery patterns (Billaut and Basset, 2007) and circadian rhythms (Giacomoni et al., 2006), but so far not with the sole purpose of comparing CE and NMT responses. One study comparing the RSA responses of youth and adults has used a truly fatiguing exercise (10 ×10 s^−1^) and has reported shorter time to reach peak power output as well as greater performance decrements (mean power output) and perceived exertion in NMT compared to CE, despite measures of power output being significantly correlated (Ratel et al., 2004). Therefore, this study aimed to compare responses to a fatiguing RSA exercise (10 ×6 s^−1^ with 24 s recovery) typically performed by practitioners in the field, using either CE or NMT, in order to establish whether both ergometers can be used interchangeably for anaerobic conditioning. Specifically, we sought to characterize whether the time course of measures of performance, fatigue, and perceived exertion was affected by ergometer type. We hypothesized that significant differences exist in the structure of performance for the same exercise performed on CE and NMT, leading to different fatigue profiles and perceived exertion.

## Methods

### Overview

The procedures described in this study were granted ethical clearance by the local ethics committee on human research (project RO1289, Bond University Research Ethics). Fourteen healthy males (age: 22.9 ± 3.1 years, height: 182 ± 5.7 cm, weight: 82.4 ± 8.5 kg) performing at least two training sessions a week were recruited in the local community using advertisements and e-mails. Participants were invited for a preliminary visit for screening against underlying health conditions potentially affecting the study procedures and findings. Inclusion in the study was conditional on providing written informed consent, and achieving a “low risk” ranking according to the ACSM guidelines, using Physical Activity Readiness (PAR-Q) and medical history questionnaires, normal (<140/90 mmHg) resting blood pressure, a body mass index <30 kg·m^2^ (BMI = body weight/height^2^), normal resting electrocardiographic activity, and normal lung function assessed using spirometry (ratio of forced expiratory volume in 1 s over forced expiratory capacity >0.70 and forced expiratory volume in 1 s >80% of predicted).

A repeated-measures design was used for this study. Participants completed four exercise sessions separated by at least 5 days to prevent from potential fatigue effects between tests. Participants reported to the laboratory after a 10- to 12-h overnight fast and no later than 10 AM to minimize the risk of hypoglycemic events and were instructed to refrain from strenuous exercise, caffeine, and alcohol in the 24 h preceding each session. The first two sessions were identical and were used to familiarize participants with RSA exercise as well as the two types of ergometers used in the study. Each session lasted ~1 h under trained personnel supervision and included two 15-min bouts of RSA separated by a 25-min recovery period, performed in either a running or a cycling modality, in a randomized order. Each bout was identical and consisted of a 10-min warm-up (5 min at an easy, constant pace with 1- to 2-s sprints every minute, followed by 5 min of dynamic stretching) followed by 5 ×6 s^−1^ RSA exercise with a 1:4 work-to-rest ratio (one 6-s sprint for every 30-s period), a shorter version of the RSA exercise used in the testing sessions.

Exercise structure for sessions 3 and 4 was identical, only differing according to the exercise modality (running vs. cycling), which was randomly assigned at session 3 for each participant, and counterbalanced for session 4. Warm-up procedures from the familiarization sessions were replicated. Participants then performed the 10 ×6 s^−1^ RSA test with the same 1:4 work-to-rest ratio. Verbal and visual cues were given for temporal feedback, and verbal encouragements were given throughout the four sessions to ensure optimal readiness and motivation.

### Procedures

**Figure 1:**
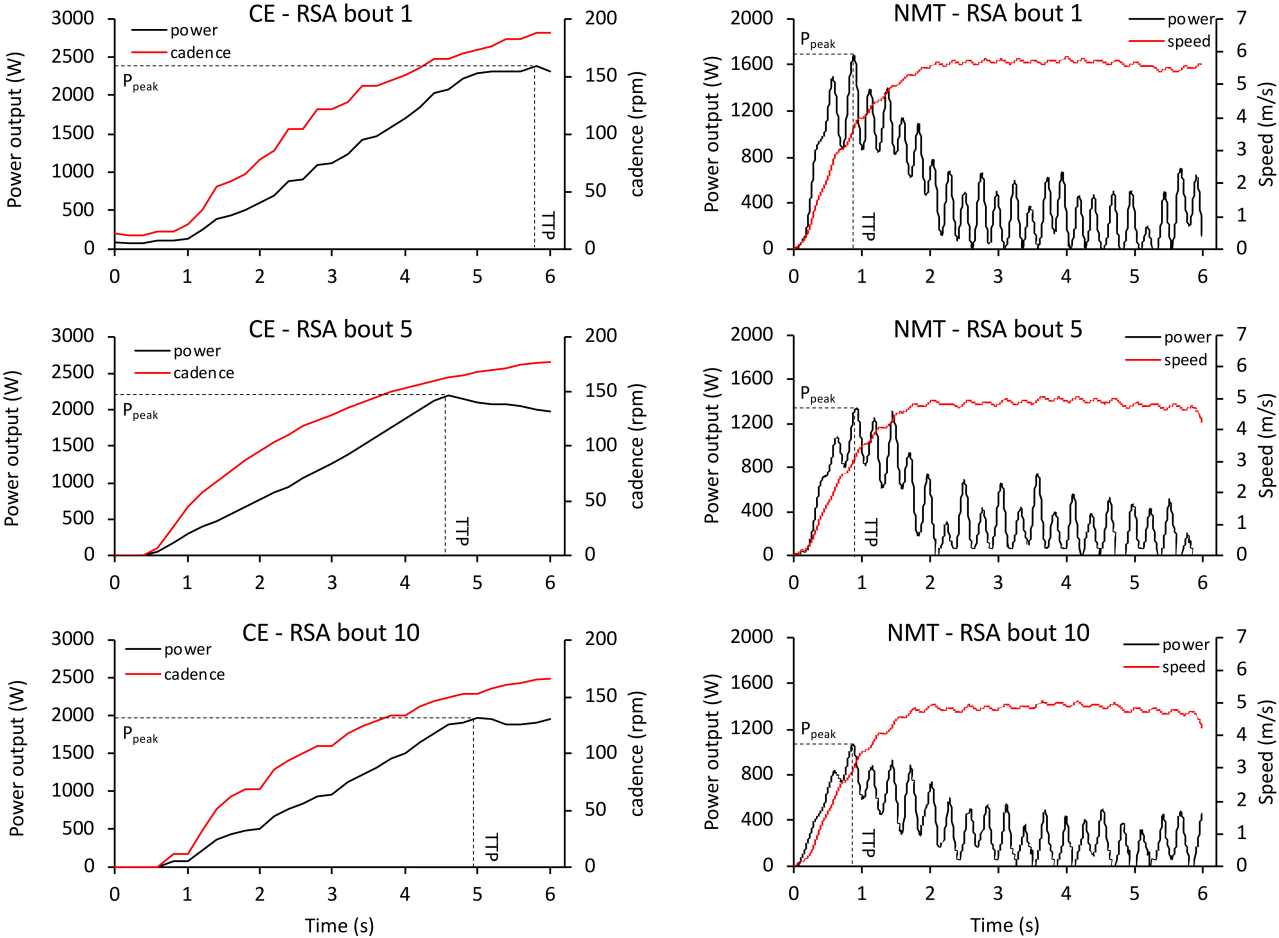
Power output as a function of time of a representative participant during the 1st, 5th, and 10th RSA bouts performed on a cycle ergometer (CE) and non-motorized treadmill (NMT).

Running RSA sessions were performed on a flat-surface NMT (Woodway, Force 3.0, USA). Treadmill belt acceleration, velocity, as well as the horizontal component of power were measured by the XPV7 PCB interface (Fitness Technology, Adelaide, Australia) and analyzed using the Force 3.0 software (Innervations Software, Joondalup WA, Australia). Running cadence (in strides per minute, spm) was then computed using the number of strides per unit time. Cycling RSA sessions were performed on an electromagnetically braked ergometer fitted with toe-clip pedals and straps (Lode Excalibur Sport, Lode BV, Groningen, The Netherlands), which provided measures of power (in W) and cadence (in rpm). The resistance (torque factor) was set at 0.70 N·kg^−1^ of bodyweight. Peak and mean power outputs (PPO and MPO) were expressed as absolute measures as well as relative to body weight, and time to reach peak power (TTP, in s) was determined for each bout (Figure 1).

Fatigue was indirectly assessed using two common approaches (Girard et al., 2011), the Fatigue Index (FI) and the decrement score (*S*_dec_), following Equations (1, 2):

(1) $$\begin{array}{r} \text{FI }(\text{\%})=100\times \left(\frac{\left[{\text{S}}_{\text{best}}-{\text{S}}_{\text{worst}}\right]}{S_{\text{best}}}\right) \end{array}$$

(2) $$\begin{array}{r} {\text{S}}_{\text{dec}}\;\left(\%\right)=\left\{1-\frac{\left(S_{1}+S_{2}+S_{3}\ldots +S_{10}\right)}{S_{best}\times n}\right\}\times 100 \end{array}$$

where *S* refers to sprint performance (PPO, in W) and *n* is the number of sprints performed.

The 6–20 linear Borg scale was used to measure ratings of perceived exertion (RPE) after the warm-up (PRE), immediately after each sprint during RSA (bouts # 1–10), and following RSA testing at 1, 3, and 5 min (POST).

### Statistical Analyses

A power analysis revealed that the study design could reliably detect significant differences between paired group means with a moderate effect size (*d* = 0.8) with a probability > 0.8, assuming a 2-sided criterion allowing for a maximum Type I error rate of α = 0.05.

Data are expressed as mean ± standard deviation (SD) and were initially tested for normal distribution using Shapiro–Wilk's *W*-test. Pearson's *r* was used to assess correlations between absolute and relative measures of power output (PPO and MPO), weight, cadence, TTP, and between the two fatigue indices (FI and *S*_dec_). Student's *t*-test was used to test for significant differences between FI and *S*_dec_ performed on CE and NMT. The main effects of exercise (bouts 1–10), ergometer (CE × NMT), and their interaction on cadence and TTP was assessed using two-way, repeated-measures ANOVAs. Initial values of RPE (PRE) on the two ergometers were compared using paired Student's *t*-test. The main effects of ergometer (CE × NMT), time of measure (RSA: bouts 1–10; recovery: bout 10, 1 min, 3 min, 5 min), and their interaction on RPE were assessed using a two-way, repeated-measures ANOVA. For all ANOVAs, Tukey's *post hoc* test was used when a significant main effect was measured to locate differences in means.

Effect sizes for pairwise comparisons were assessed using Cohen's *d*, calculated in the standard manner and interpreted according to Cohen's scale (small effect: 0.2 < *d* < 0.5, medium effect: 0.5 < *d* < 0.8, and large effect: *d* > 0.8). For ANOVAs, we reported effect size using partial eta-squared (η^2^_*p*_) interpreted according to Cohen's scale (small effect: 0.01 < η^2^_*p*_ < 0.06, medium effect: 0.06 < η^2^_*p*_ < 0.14, and large effect: η^2^_*p*_ > 0.14). All statistical analyses were performed using the open-access statistical package jamovi (Jamovi project, 2018), and the level of significance set at *p* < 0.05.

## Results

### Performance Measures

**Figure 2:**
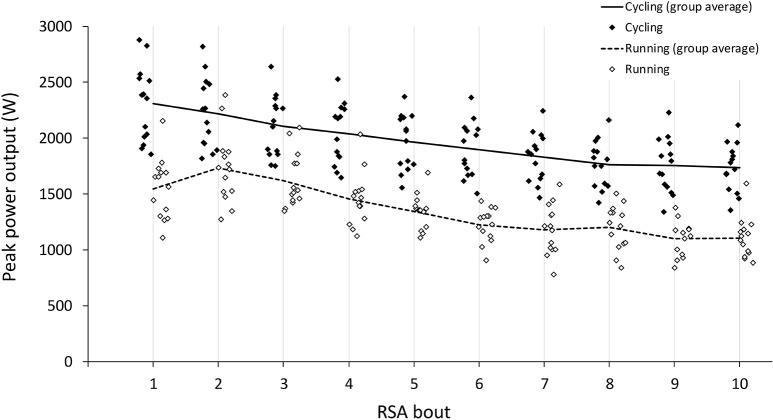
Individual and group average peak power outputs achieved during the bouts of 10 ×6 s^−1^ RSA test performed on a cycle ergometer (CE) and non-motorized treadmill (NMT).

**Figure 3:**
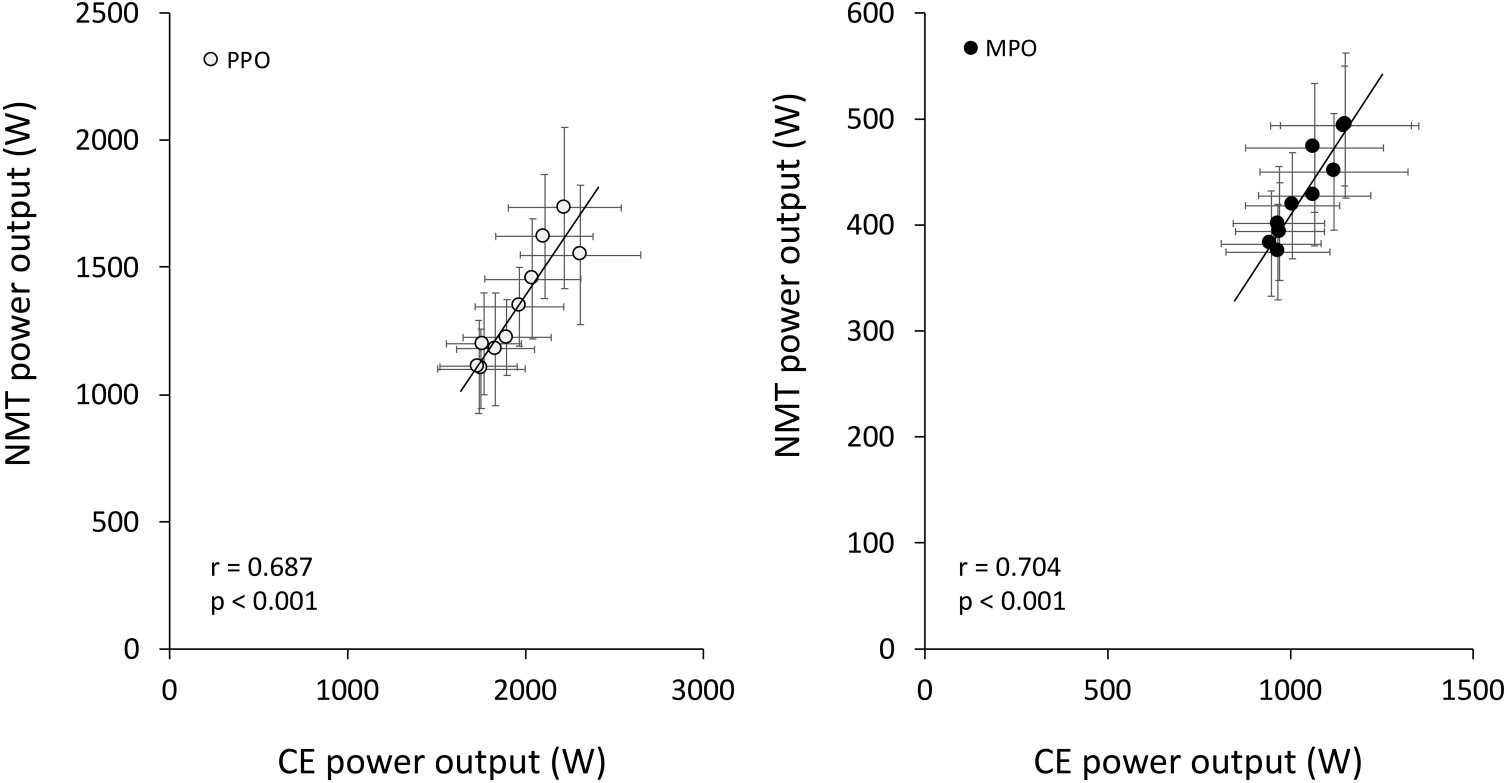
Relationship between peak (PPO) and mean power output (MPO) achieved during the bouts of 10 ×6 s^−1^ RSA test performed on a cycle ergometer (CE) and non-motorized treadmill (NMT). Pearson's correlation coefficient *r* and associated *p*-value are also reported.

All 14 participants completed the study. Absolute individual and group mean PPO profiles during CE and NMT tests are presented in Figure 2, and absolute and relative MPO and cadence are presented in Table 1. Participants' weight was significantly correlated with absolute PPO and MPO on CE (PPO: *r* = 0.900, *p* < 0.001; MPO: *r* = 0.792, *p* < 0.001) and only with MPO on NMT (PPO: *r* = 0.337, *p* = 0.238; MPO: *r* = 0.840, *p* < 0.001). Measures of power output on CE and NMT were significantly correlated for absolute PPO and MPO (Figure 3), but not for relative measures (PPO: *r* = 0.497, *p* = 0.070; MPO: *r* = 0.339, *p* = 0.235).

**Table 1:** Descriptive measures of absolute and relative mean power output (MPO) and cadence achieved during the 10 ×6 s^−1^ RSA test on CE and NMT.

	**CE**	**CE**	**CE**	**NMT**	**NMT**	**NMT**
**Bouts**	**MPO**	**MPO**	**Cadence**	**MPO**	**MPO**	**Cadence**
	**(W)**	**(W·kg^**−1**^)**	**(rpm)**	**(W)**	**(W·kg^**−1**^)**	**(spm)**
1	1,152 ± 179	14.0 ± 1.4	112 ± 9	494 ± 68	6.0 ± 0.7	230 ± 16
2	1,149 ± 203	13.9 ± 1.6	112 ± 9	493 ± 57	6.0 ± 0.4	221 ± 13
3	1,066 ± 189	12.9 ± 1.5	106 ± 12	473 ± 61	5.7 ± 0.5	213 ± 11
4	1,119 ± 202	13.5 ± 1.5	111 ± 12	450 ± 55	5.5 ± 0.4	204 ± 11
5	1,067 ± 154	13.0 ± 1.4	107 ± 10	428 ± 48	5.2 ± 0.3	197 ± 11
6	1,006 ± 129	12.2 ± 1.2	102 ± 8	418 ± 50	5.1 ± 0.3	191 ± 13
7	909 ± 125	11.8 ± 1.4	99 ± 9	401 ± 54	4.9 ± 0.4	186 ± 16
8	972 ± 122	11.8 ± 1.4	100 ± 8	394 ± 46	4.8 ± 0.4	182 ± 15
9	966 ± 142	11.7 ± 1.1	100 ± 8	374 ± 45	4.5 ± 0.3	176 ± 18
10	948 ± 137	11.5 ± 1.4	98 ± 10	382 ± 50	4.6 ± 0.4	183 ± 13
**AVG**	**1,041** **±** **141**	**14.4** **±** **1.5**	**105** **±** **8**	**431** **±** **48**	**6.2** **±** **0.6**	**198** **±** **8**

**Figure 4:**
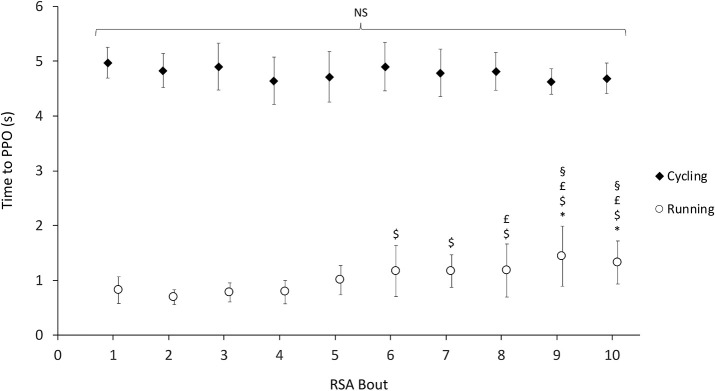
Time to peak power output (PPO) as a function of bout number during the 10 ×6 s^−1^ RSA test. Symbols *, $, £, and § denote significant differences compared to bouts 1, 2, 3, and 4, respectively (*p* < 0.01).

Cadence was significantly higher on NMT compared to CE (*p* < 0.001; η^2^_*p*_ = 0.982), with significant effects of exercise (*p* < 0.001; η^2^_*p*_ = 0.822) and interaction (*p* < 0.001; η^2^_*p*_ = 0.605). *Post hoc* tests revealed that cadence significantly decreased only at the seventh bout and stabilized until the end of the RSA test, whereas it decreased throughout the RSA test on NMT. Cadence was significantly correlated with absolute power output on CE (PPO: *r* = 0.736, *p* = 0.003; MPO: *r* = 0.884, *p* < 0.001), but not on NMT (PPO: *r* = 0.014, *p* = 0.952; MPO: *r* = −0.029, *p* = 0.920). Time to reach PPO was significantly shorter on NMT compared to CE (*p* < 0.001; η^2^_*p*_ = 0.996), with significant effects of effect of exercise (*p* < 0.001; η^2^_*p*_ = 0.220) and interaction (*p* < 0.001; η^2^_*p*_ = 0.379) (Figure 4). *Post hoc* tests revealed that TTP increased on NMT from bout #1 to #9 and 10; from bout #2 to #6, 7, 9, and 10; from #3 to #8, 9, and 10; and from #4 to #9 and 10, with no significant changes for CE (Figure 4). Time to reach PPO was inversely correlated with cadence on CE (*r* = −0.565, *p* = 0.035) and NMT (*r* = −0.669, *p* = 0.009).

### Fatigue Indices

Each participant achieved their highest PPO during the first RSA bout on CE, whereas they achieved it during the first (*n* = 3), second (*n* = 7), and third bout (*n* = 4) on NMT (Figure 2). Indices of fatigue FI and *S*_dec_ were significantly greater on NMT compared to CE, with large effect size (Table 2). On CE, significant correlations existed between *S*_dec_ and absolute (*r* = 0.557, *p* = 0.038) and relative PPO (*r* = 0.578, *p* = 0.030), and there were no significant correlations between FI and measures of power output. On NMT, significant correlations existed between FI and absolute (*r* = 0.634, *p* = 0.015) and relative PPO (*r* = 0.817, *p* < 0.001), and between *S*_dec_ and absolute (*r* = 0.690, *p* = 0.006) and relative PPO (*r* = 0.870, *p* < 0.001). There were no significant correlations between FI or *S*_dec_ and absolute or relative MPO on CE.

**Table 2:** Measures of fatigue index and decrement score achieved during the 10 ×6 s^−1^ RSA test.

	**CE**	**NMT**	**Student's *t***	***p*-value**	**Cohen's *d***
Fatigue Index (%)	25.1 ± 5.7	39.0 ± 10.5	−4.59	<0.001*	−1.23
Decrement Score (%)	14.7 ± 3.6	22.6 ± 7.3	−4.08	0.001*	−1.09

Student's t statistic and associated p value and effect size (Cohen's d) are also reported. The symbol * denotes significant differences.

### Perceived Exertion

**Figure 5:**
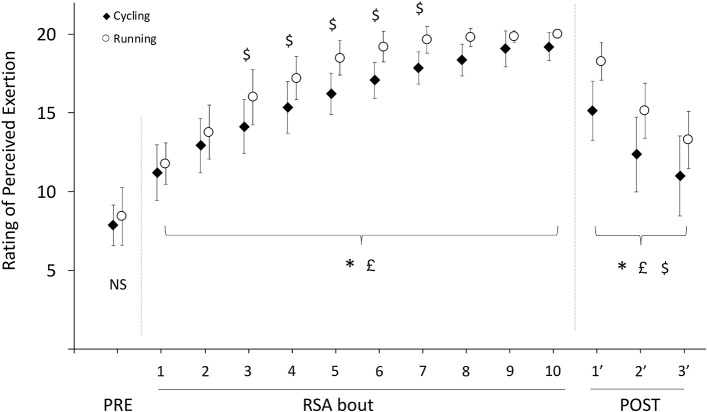
Ratings of perceived exertion during the 10 ×6 s^−1^ RSA test. Symbols *, £, and $ denote significant main effects of exercise, ergometer, and interaction, respectively (*p* < 0.05).

There were no significant differences in RPE for CE (7.9 ± 1.3) and NMT (8.4 ± 1.8) before RSA (*p* = 0.385; *d* = −0.24). During RSA, there were significant and large practical effects of exercise (*p* < 0.001; η^2^_*p*_ = 0.952), ergometer (*p* < 0.001; η^2^_*p*_ = 0.694), and interaction (*p* < 0.001; η^2^_*p*_ = 0.252). *Post hoc* tests revealed that the overall significant increase in RPE leveled off after the fifth bout, and that the increase in RPE was significantly greater on NMT compared to CE from bouts 3 to 7 (Figure 5). Maximum RPE was reached at the 10th bout of exercise on both CE and NMT (19.2 ± 0.9 vs. 20.0 ± 0.0, respectively), with 20/20 scoring attained on NMT for the 14 participants (as early as bout 4) and 20/20 scoring attained on CE only for six participants. During recovery, there were significant and large effects of time (*p* < 0.001; η^2^_*p*_ = 0.930), ergometer (*p* < 0.001; η^2^_*p*_ = 0.600), and interaction (*p* < 0.001; η^2^_*p*_ = 0.349). *Post hoc* tests revealed that RPE was consistently higher on NMT compared to CE at all times during recovery (Figure 5).

## Discussion

The main finding of this study was that a 10 ×6 s^−1^ RSA exercise was associated with markedly different outcomes in performance, fatigue, and perceived exertion when performed on CE or NMT. We observed that (i) absolute power output (PPO and MPO) on the two ergometers were correlated, and predicted to some degree by body weight, (ii) peak power output was achieved early on NMT and late on CE during each bout, leading to “frontloaded” and “backloaded” power output profiles, (iii) the associated fatigue indices were greater on NMT compared to CE, and (iv) perceived exertion increased faster and to a greater extent on NMT compared to CE. Together, these findings provide further insights for sport scientists and practitioners to evaluate ergometer specificity requirements for testing and exercise prescription and programming.

In the current study, we reported a strong agreement between absolute measures of PPO and MPO on the two ergometers, indicating that NMT may be used to profile athletes based on absolute, but not relative power during RSA. In agreement with previous research using a fatiguing RSA exercise (Ratel et al., 2004), we also observed that the faster TTP of RSA on NMT (and in our study, also a higher cadence) was associated with increased fatigue and perceived exertion compared to CE. Although we have not quantified it on NMT, this frontloaded performance profile obviously originates in a comparatively lower resistive torque setting than on CE allowing for faster acceleration. Such factory settings are used by practitioners and scientists as they mimic overground sprinting accurately (Carling et al., 2012; Nédélec et al., 2013; Aldous et al., 2014) and are unlikely to be modified since estimating optimal resistive load to maximize power output is a complex athlete- and equipment-specific procedure. As was the case for NMT, we selected the resistive torque for CE in the current study (0.70 N·kg^−1^) based on values typically used by practitioners and scientists (Chia and Lim, 2008; Gonzalez et al., 2013). Although higher resistive torques are typically used with power athletes during single sprints and have been used for RSA testing (Billaut et al., 2011), a 0.70 N·kg^−1^ load could even be argued to correspond to the lower end of the recommended spectrum for reliable PPO generation over repeated sprints (Bogdanis et al., 2008), especially in power-based athletes such as the participants of the current study. Even lower resistive torques have also been used to minimize the work over inertia during the initial push phase (Doré et al., 2000).

Further, participants in the current study achieved the highest PPO in the second or even the third bout on NMT, while they all achieved it during the first bout on CE, indicating that they may have regulated their efforts differently on NMT and CE. Pacing is likely to have occurred despite participants being habituated to the procedures and instructed to give maximum effort on each bout, as it has been previously observed even during RSA (Billaut et al., 2011). However, several factors are converging against the simple explanation that a more conservative pacing strategy characterizes RSA performed on NMT, such as similar dynamics in MPO decrease compared to CE (from the first bout onwards), together with a more rapid increase in RPE and higher fatigue indices on NMT. Unfortunately, we profiled participants' maximum speed only on NMT for monitoring habituation effects, and we have not profiled participants' maximum power during a single sprint on CE. Still, during RSA on NMT, participants achieved 101.1 ± 4.2% of their individual maximum velocity achieved during a single bout, which indicates that participants were not only sufficiently habituated to NMT, but were also likely to be performing to the best of their abilities. Overall, this indicates that factors other than pacing could underpin the differential dynamics in PPO observed on NMT and CE. Importantly, previous research having used a light resistive torque (50 g·kg^−1^) during RSA have also reported similar performance decrements on NMT and CE for MPO, but not for PPO (Ratel et al., 2004). Therefore, one of the main questions sports practitioners should be asking themselves is whether they want to minimize differences between ergometers (either by increasing resistive torque on NMT or by reducing it on CE) or to maintain real-world applicability by selecting resistive torques typically used on each ergometer.

Therefore, it is paramount for practitioners and applied scientists to recognize that testing and training for RSA on one mode of exercise may not be relevant for the other mode. Of particular importance for a weight-bearing activity like running (including on a treadmill) was that body weight and cadence were not significant predictors of power output for NMT, unlike what we observed on CE. One of the most striking results was that group average PPO increased overall in the second bout despite alterations in cadence (and for several participants, in the third bout), suggesting that power output was potentiated in NMT (unlike in CE). The origin of such discrepancies in predictors of performance could partly originate in exercise mode specificity, with additional support and transport of body mass on NMT, which incurs a greater metabolic work than on level cycling and CE. In support of these observations, previous studies (Young et al., 1995; Cronin and Hansen, 2005; Markström and Olsson, 2013) have found that relative strength and power are important components of run-based sprint performance in contrast to cycling-based sprinting, where absolute muscle mass, strength, and power are the best predictors of performance (Millet et al., 2009; Rønnestad et al., 2010). As such, practitioners should use caution when interpreting or comparing absolute and relative power profiles derived from NMT and CE testing, and use the ergometer that yields the variable of greatest interest for planning the development of physical abilities in their athletes. Methodologically, our study also reinforces the notion that *S*_dec_ is a superior metric compared to FI for running-based RSA as it takes into account the performance of every RSA bout (Girard et al., 2011), and therefore may capture more accurately non-linear variations in performance.

Other mechanisms are likely at play to explain the observed differences in performance, fatigue, and perceived exertion between ergometers and should be mentioned. These could be of metabolic and neuromuscular origins. Firstly, the larger active muscle mass involved in running compared to cycling during intense exercise (Millet et al., 2009) may have elicited a greater metabolic response (ATP turnover), and thus a greater reliance on glycolytic energy pathway to compensate for limitations in aerobic metabolism to provide sufficient energy in the later bouts of repeated sprints (Girard et al., 2011), as indirectly evidenced by the greater alterations in power and cadence, the increased TTP from 6 to 10th bouts observed only in NMT, as well as the higher perceived exertion during and after the RSA test. Secondly, the larger active muscle mass recruited during running compared to cycling could also lead to greater central drive limitations (Rossman et al., 2014) and contribute to explain the different outcomes on performance, fatigue, and perceived exertion variables measured in the current study between the two ergometers. Although several factors have been identified as possible mechanisms for the development of neuromuscular fatigue during repeated sprints (Girard et al., 2011), alterations in central (motor) drive during RSA could at least partly originate in increased group III and IV afferent feedback associated with the development of peripheral acid–base imbalance caused by the reliance on anaerobic glycolysis (Siegler and Marshall, 2015). As such, alterations in power output, cadence, TTP, and perceived exertion measured in the current study could be indicative of the onset of central fatigue, as has been suggested by similar observations in previous research on RSA (Goodall et al., 2015; Pearcey et al., 2015; Hureau et al., 2016). Such differences in the etiology of fatigue in running and cycling have been observed following RSA, where the maximum force of knee extensors decreased throughout a 5 ×6 s^−1^ RSA running exercise with accompanying losses in central activation, and decreased only after the last repeated bout in cycling RSA with no central activation losses (Tomazin et al., 2017). Finally, the specificity of contraction patterns in running and cycling modes could account for some of the observed differences we observed in the current study. For instance, it has been recently reported that cycling elicited similar or even higher levels of peripheral fatigue compared to running during high-intensity sprint exercise, differences likely originating from the longer fractional contraction duration phases in cycling limiting local blood flow (Rampinini et al., 2016). Differences in contraction patterns in running and cycling have also been shown to influence the relative aerobic and anaerobic energy contributions to exercise performed in the severe intensity domain, affecting differentially the characteristics of the power–duration relationship in running and cycling (Weyand et al., 2006).

Overall, while it is possible that differences in inertial load between ergometers may have blunted participants' “true” peak power capacity, as was previously observed in CE (Bogdanis et al., 2008), fatigue has been shown to remain relatively unaffected at lower inertial loads on CE (Bogdanis et al., 2008), such as those used in the current study. Therefore, it is likely that differences in fatigue profiles, in relative energy contribution, and in force application patterns are explaining most of the differences in power output, cadence, TTP, performance decrements, and perceived exertion across the two ergometers for the same 10 ×6 s^−1^ RSA exercise. Since we have not directly assessed the etiology of fatigue or relative energy systems contributions in the current study, further research is required to ascertain the origin of performance discrepancies between similar RSA exercise performed using two exercise modes.

The current study had several limitations. Mainly, we did not measure energy pathway contribution or actual neuromuscular function alterations (strength loss of a muscle group), which, as we discussed, could both yield important findings to further our comprehension of the task specificity of RSA. Ultimately, understanding the origins of performance limitations, including pacing, during RSA could yield important insights for optimizing training and conditioning strategies. Additionally, the measure of absolute PPO used in this study is specific to the brand and make of CE, and measures of PPO may appear high compared to other models as it has very high resolution (one measurement every 2°) and performed on young healthy university-aged participants. These results may thus not be directly comparable to other brands and makes, and to other participant groups. Our rationale was that PPO is typically used by practitioners for player profiling and should therefore provide the basis of our analysis. Finally, future research should also aim to quantify resistive torque on NMT, since, as we pointed out, scientific research has so far not been able to provide field practices with simple, actionable guidelines for specific conditioning. For instance, using athlete-specific resistive torque tailored using individual force–velocity profiling has the potential to guide exercise prescription more precisely and effectively than the current, one-size-fits-all approach.

## Conclusion

The findings of the current study indicate that RSA performed on a NMT induces greater alterations in fatigue and physiological load than cycling ergometry, as evidenced by the greater fatigue indices and perceived exertion elicited by the running mode. While future research should be performed to ascertain the origin of this additional load compared to cycling, these results indicate that testing and training for power on one mode of exercise may not be relevant for the other mode. Therefore, coaches should cautiously appraise ergometer specificity for exercise prescription, taking into account recovery needs and adverse effects of fatigue.

## Data Availability Statement

The anonymized datasets generated for this study are available at the following repository: https://doi.org/10.6084/m9.figshare.11961159.v1.

## Author Contributions

HK and DS were responsible for preparing the manuscript. DS, CM, and DL were responsible for data collection. HK, DS, and DL performed the data analysis. DL and CM supervised the study. All authors contributed to the concepts and framework presented in the manuscript and to the revision of the manuscript, and approved the submitted version.

## Conflict of Interest

The authors declare that the research was conducted in the absence of any commercial or financial relationships that could be construed as a potential conflict of interest.
